# Substance Use as a Strong Predictor of Poor Academic Achievement among University Students

**DOI:** 10.1155/2017/7517450

**Published:** 2017-06-07

**Authors:** Tesfa Mekonen, Wubalem Fekadu, Tefera Chane Mekonnen, Shimelash Bitew Workie

**Affiliations:** ^1^Psychiatry Department, College of Health Sciences and Medicine, Bahir Dar University, Bahir Dar, Ethiopia; ^2^College of Health Sciences and Medicine, Wollo University, Dessie, Ethiopia; ^3^School of Public Health, College of Health Sciences and Medicine, Wolaita Sodo University, Wolaita Sodo, Ethiopia

## Abstract

**Background:**

Substance use is a growing concern globally and its association with students' academic performance is not well studied.

**Objective:**

This study was aimed to assess the prevalence of substance use (alcohol, tobacco, and khat) and its association with academic performance among university students.

**Methods:**

Cross-sectional study was conducted among Wolaita Sodo University students. A total of 747 students were selected by using cluster sampling technique. Data were collected by pretested self-administered questionnaire and examined using descriptive statistics and linear regression with 95% confidence intervals. Variables with *p* value of less than 0.05 were considered as statistically significant.

**Result:**

Prevalence of substance use (alcohol, tobacco, and khat) was 28.6%. Substance use (current smoking, chewing khat at least weekly, drinking alcohol on a daily basis, and having intimate friend who uses substance) was significantly and negatively associated with students' academic performance.

**Conclusion:**

Substance use among Wolaita Sodo University students was as common as other studies in Sub-Saharan countries and negatively associated with students' academic achievement. The common practice of substance use and its association with poor academic performance demand the universities to have a good control of substance and to implement youth friendly activities.

## 1. Background

According to world health organization, psycho active substance is any chemical substance which alters physical and/or mental function. Substance abuse is also defined by DSM-V as use of any drug, usually by self-administration, in a manner that deviates from approved social or medical patterns [1, 2].

For different reasons, substance use has been practiced by students regardless of the negative consequences [3]. Use of psycho active substances such as khat (*Catha edulis*, which is evergreen leaf common in east Africa), tobacco products (cigarette, shisha), and alcoholic beverages have become major public health challenge [4].

Student substance use is associated with different factors like sex, age, peer pressure, family substance abuse, personal pleasure, and poor academic achievement [3, 5, 6]. According to the national youth risk behavior survey, USA, negative association was seen between alcohol and other drug use and academic achievement after controlling for sociodemographic variables [7]. Another study conducted in USA school adolescents indicated that students who were engaged in different substance use practices were poor academic achievers [8]. A study conducted at Jimma University also showed that substance use was associated with poor academic achievement [9].

Despite some researchers suggesting that substance use is associated with poor academic performance, many students used variety of substances for the sake of good academic achievement [3, 10]. As a study conducted in Turkey indicates, substance use was practiced by significant proportion of university students; cigarette was smoked by 24.8% students regularly every day; alcohol was drunk occasionally by 37.8% of university students and by 8.1% of students regularly [11]. In Iran substance (alcohol, cigarette, and water pipe) was used by 22% male medical students and 8% female medical students at least once in their life [12]. Alcohol was consumed daily by 42% of Osun State University students in Nigeria and 34.1% of students consumed alcohol weekly. As a research done in South Africa showed the occurrence of alcohol use was 38.7%, tobacco smoking 30%, and cannabis was 8.4% [13].

Different substances like khat, alcohol, and tobacco products have been consumed by significant proportions of university students in Ethiopia. A cross-sectional study among undergraduate medical students of Addis Ababa University indicated that the lifetime prevalence of khat user was 14.1% [4]. The prevalence of khat chewing among Axum University students, North Ethiopia, was 27.9% [14]. According to the study conducted among eastern Ethiopian school adolescents, study conducted in undergraduate medical students of Addis Ababa University and Axum University students, the lifetime prevalence of cigarette smoking was 12.4%, 8.7%, and 9.3%; from those 4.2%, 1.8%, and 9.3% were daily smokers, respectively [4, 14, 15]. The research done in eastern Ethiopia showed the lifetime prevalence of cigarette smoking among school adolescents as 12.2% [16].

Since the proportion of student population in higher educational institutions is increasing, substance use and its association with academic performance is going to be an increased concern. As a good alert for universities and policymakers about the effect of substance use on academic performance; the result of this study will play a paramount role to conduct interventional measures with evidence based actions. Hence, the main purpose of this study was to assess the prevalence of substance use and its association with academic achievement among university students.

## 2. Method

Cross-sectional study was conducted at Wolaita Sodo University from February 10 to 20, 2015. Wolaita Sodo University is located in the mid highlands of south Ethiopia. Specifically, it is found in Wolaita Zone, with coordinate (6°49′N latitude and 37°45′E longitude) which lies approximately 330 KM south of Addis Ababa.

The sample was calculated by using single population proportion formula. A 32.8% rate of substance use was taken from a study conducted at Axum University students with a margin of error 5%, confidence level 95%, and nonresponse rate of 10%. By considering design effect of the two, the final sample size was 747.

The study participants were recruited by cluster sampling method. Each department (section) from each college and school regardless of their academic year were listed and finally the section was taken as cluster which was selected randomly by lottery method.

The variables studied under this study were academic performance which was the dependent variable and the main independent variable was students' substance use. Other covariates were socioeconomic status of the students, peer pressure, origin of residence, any chronic illness, year of study, and parents' substance use.

For the sake of this research, substance use was defined as using either khat (*Catha edulis*), tobacco products or alcoholic beverages or any combination of those substance. When the study participants used substances (alcohol, chat, or tobacco) even once in their life, it was defined as lifetime use. Current use of substance is when the study participants were using those substances in the last 12 months from the data collection time [17, 18].

The data were collected by pretested self-administered questionnaire by well-trained data collectors. The questionnaire for substance use was adopted from WHO ASSIST [16, 17]. Academic achievement was assessed by students' CGPA (Cumulative Grade Point Average) scores at the end of the semester which were reported by the students. The students' CGPA is based on the Ethiopian higher education grading system which is scored out of 4 points. The questionnaire for sociodemographic and other variables was prepared from reviewed literatures.

The collected data were entered into Epi-Info version 3.5.2 and then transported to SPSS version 21 for further statistical analysis. Descriptive statistics were used for presenting the descriptive results. *T*-test with respective *p* value was used to show the mean differences in CGPA. The variables were checked for uniformity, outliers, and multicollinearity, and the variance was normally distributed. Pearson correlation was used to see the correlation of substances with students' grade point. Those variables with *p* value of less than 0.2 in univariate analysis were included and analyzed by multiple linear regression with 95% confidence interval. Standardized and unstandardized regression coefficients were used to indicate the associations. Variables with *p* values < 0.05 were considered as statistically significant.

The ethical approval was obtained from Research Ethics Review Committee of College of Health Sciences and Medicine, Wolaita Sodo University. The purpose and importance of the study were explained for the participants and written consent was obtained from each participant. Confidentiality was maintained by anonymous questionnaire and by keeping the data in secure place.

## 3. Results

### 3.1. Sociodemographic Characteristics of the Study Participants

From the selected 747 participants 725 were involved in the study which makes the response rate 97.05%. Among the total 725 participants, 482 (66.5%) were male and the mean age was 21.18 years (SD ± 1.79 years). From the total participants 343 (47.3%) were protestant in their religion, 585 (80.7%) were single in their marital status, and 191 (26.3%) were Wolaita in their ethnicity. The reported monthly pocket money of the participants was ranged from 0 to 3600 Ethiopian birr with median value of 200 Ethiopian birr (Table 1).

### 3.2. Characteristics of Students' CGPA

The Cumulative Grade Point Average of the students was ranged from 1.70 to 3.98. The mean score of students' CGPA was 2.93 (±0.52). From the total students who participated in the study, 240 (33.1%) of them had used at least one substance in their lifetime. The mean difference of CGPA with gender is statistically significant (*p* value < 0.001). The mean differences of CGPA with substance related variables were also statistically significant (Table 2).

### 3.3. Substance Use Characteristics of the Students

From the total students who participated in the study, 207 (28.6%) of them used at least one substance in the last 12 months since the data collection time; and from those who use substance, 86.5% of them uses alcohol, 35.7% use khat, and 19.8% use tobacco products. From the total students who participated in the study, 24.7% of them use alcohol, 10.2% of them use khat, and 5.7% of them use tobacco products in the last one year since the data collection time. More than half of substance users started to use substance in their high school life before joining university (Table 3).

### 3.4. Correlation of Selected Variables with Students' Cumulative Grade Point Average

Using different substances like tobacco, khat, and alcoholic beverages had negative correlation with students CGPA (Table 4).

### 3.5. Association of Academic Performance with Substance Use and Other Predictors

The final model of multiple linear regression explained 45% of the variation in reported Cumulative Grade Point Average (CGPA) of the students. The variables with significant association to academic performance were male gender (B = 0.35, 95% CI: 0.19, 0.52), urban origin of residence (B = −0.19, 95% CI: −0.32, −0.06), current smoking (B = −0.27, 95% CI: −0.46, −0.09), chewing khat at least weekly (B = −0.24, 95% CI: 0.44, −0.04), having intimate friend who uses substance (B = −0.17, 95% CI: −0.31, −0.03), and drinking alcohol on a daily basis (B = −0.51, 95% CI: −0.79, −0.23) (Table 5).

## 4. Discussion

This study clearly indicated that substance use is becoming a huge concern among university students. The overall prevalence of substance use among Wolaita Sodo University students was 28.6%. The most frequently consumed substance by the students was alcohol, followed by khat and tobacco products, respectively. This finding was consistent with the study done in Southern Iran but lower than the study conducted in Northern Ethiopia [3, 12]. Alcohol was consumed by 24.7% of the students and this was consistent with the study conducted in different higher education institutions in Ethiopia; it was 21.6% among Addis Ababa University medical students, 20% in Haramaya University students, and 21.7% in college students of Southern Ethiopia [19, 20]. However, the finding in this study was lower than the study conducted among Axum University students in which the alcohol consumption was 32.8%. In addition, the finding of this study was much more less than the study conducted in Trinidad and Tobago university students in which the six-month alcohol consumption was 70%, and Turkey University students in which it was 37.9% [11, 14, 21]. This might be due to cultural and socioeconomic differences.

The prevalence of khat use was 10.2% and this finding was in line with the study conducted among medical students of Addis Ababa University which was 7% [4]. But the finding in this study was lower than the findings from Haramaya University, 23.6%, and elsewhere in Southern Ethiopia, 27.7% [19, 20]. The possible explanation for the observed difference might be due to environmental conditions like the availability of the specific substance.

Tobacco products were used by 5.7% of the students and it was consistent with other studies done among University students in Ethiopia [16]. However, the finding was lower as compared with the study done among Haramaya University students which was 10.8% and Italian University students in which current smoking was 24% [19, 22].

The use of substance is known for its significant association with mental distress and consequently this mental distress can affect the students' academic performance negatively [23–25]. As the study conducted at Jimma university students indicated, substance use was associated with low academic performance [9]. This may be due to the consequences of substance use towards social, economic, physical, and psychological aspects of the student. There may be conflict with parents or friends, health problems, financial hardships, and emotional disturbance because of substance use [26, 27].

Students who were current smoker had significantly high risk of poor academic performance (lower score of Cumulative Grade Point Average) as compared with nonsmokers. Smoking is known to be a getaway substance for other illicit drug uses, high risk drinking behavior, and high risk sexual behavior as well. Those complex interactions with other risky behaviors make smoking the important predictor of poor academic performance [6].

Khat chewing was also associated significantly and negatively with academic performance of the students. The reported CGPA of the students who were chewing khat at least weekly was less than those who were not chewing khat at all. Even though most students perceived that khat use improves academic performance [28], it was associated with poor CGPA score as indicated by this study. Khat chewing usually tends to be more ceremonial activity and most of the khat sessions took long durations followed with smoking and drinking alcohol, which may interfere with students' academic activities negatively [29, 30].

Drinking alcoholic beverages was also significantly and negatively associated with the academic performance. Those who were drinking on a daily basis had scored significantly lower CGPA than those who never drank alcoholic beverages in the last 12 months. Alcohol clouds judgment and can make the student be careless about academic and other success issues. This finding was consistent with the study done in United Kingdom University students in which alcohol consumption was negatively associated with academic performance [31]. In the study conducted elsewhere among adolescents, academic difficulties associated with alcohol consumption were reported [2].

Not only using substances was associated with academic performance, but also having an intimate friend who uses substance was associated. Peer pressure is known to be an important predictor to shape adolescents behavior either in good or bad way. The CGPA of students who have intimate friend who uses substance was significantly lower as compared with those who did not have intimate friend who uses substance. This finding was supported by the research done in Ethiopia among higher institution students in which peer pressure was one of the factors associated with students' substance use [3, 16].

The other predictors with significant association with students' academic performance were sex and residence, in which male students scored significantly higher CGPA than females and the CGPA of students from urban area was less than that of the students from rural area. Though evidence from developed countries reports that female students' academic performance is better as compared with males [32, 33], this is not the case in resource limited setting where female enrollment and academic performance is in lower rate. Communities' attitude for female education, higher gender based violence and disproportionate cultural, and socioeconomic and psychological burdens in female students might contribute to poor academic achievement of female students [34–36].

## 5. Conclusion and Recommendations

Substance use was high among Wolaita Sodo University students. Current smoking, khat chewing, drinking alcohol, having intimate friend who uses substance, and urban residence were significantly associated with poor CGPA score. The high prevalence of substance use and its association with poor academic performance demand from the universities to have a good control of substance and to implement youth friendly activities to bring behavioral change on their students. It is also better to empower female students by making them active participants in class as well as in teaching learning activities in order to increase their CGPA.

## Figures and Tables

**Table 1:** Sociodemographic characteristics of Wolaita Sodo University students, 2015.

Variable	Frequency (*n*)	Percent (%)
Sex		
Male	482	66.5
Female	243	33.5
Religion		
Protestant	343	47.3
Orthodox	291	40.1
Muslim	78	10.8
Catholic	13	1.8
Marital status		
Single	585	80.7
In open relationship	99	13.7
Married	30	4.1
Divorced and widowed	11	1.5
Monthly pocket money		
≤200 ETB	207	28.6
>200 ETB	518	71.4
Ethnicity		
Wolaita	191	26.3
Oromo	154	21.2
Amhara	107	14.8
Gamo	96	13.2
Sidama	63	8.7
Gurage	57	7.9
Hadya	27	3.7
Others^*∗*^	30	4.2
Origin of residence		
Rural	412	56.8
Urban	313	43.2
Year of study		
First year	192	26.5
Second year	221	30.5
Third year	166	22.9
Fourth year	82	11.3
Fifth year	64	8.8

Ethnicity, others^*∗*^: siltie, tigre, and kefa; ETB: Ethiopian Birr.

**Table 2:** Mean difference of CGPA with respect to substance use and other selected variables, 2015.

Variables	Frequency *n* (%)	CGPA mean (SD)	*t*	*p* value
Gender				
Male	482 (66.5)	3.05 (0.48)	8.55	<0.001
Female	243 (33.5)	2.72 (0.49)		
Age group				
Below mean age	454 (62.6)	2.91 (0.52)	−1.38	0.17
Above mean age	271 (37.4)	2.97 (0.51)		
Residence				
Rural	412 (56.8)	2.96 (0.51)	1.69	0.09
Urban	313 (43.2)	2.89 (0.52)		
Parent substance use				
Yes	160 (22.1)	2.92 (0.57)	−0.46	0.65
No	565 (77.9)	2.94 (0.49)		
Lifetime substance use				
Yes	240 (33.1)	2.82 (0.55)	−4.47	<0.001
No	485 (66.9)	2.99 (0.48)		
Substance abuse^*∗*^				
Yes	98 (13.5)	2.64 (0.57)	−6.15	<0.001
No	627 (86.5)	2.98 (0.49)		
Current alcohol				
Yes	179 (24.7)	2.78 (0.58)	−4.74	<0.001
No	546 (75.3)	2.98 (0.48)		
Current khat				
Yes	74 (10.2)	2.56 (0.51)	−6.67	<0.001
No	651 (89.8)	2.97 (0.50)		
Current smoking				
Yes	41 (5.7)	2.34 (0.41)	−7.84	<0.001
No	684 (94.3)	2.97 (0.50)		
Hostel user				
Yes	518 (71.4)	2.98 (0.51)	3.95	<0.001
No	204 (28.6)	2.81 (0.51)		

SD: standard deviation. ^*∗*^based on CAGE-AID score.

**Table 3:** Substance use and some selected variables of students with respect to gender, 2015.

Variables	Gender		*χ* ^2^	df	*p* value
	Male *n* (%)	Female *n* (%)			
Alcohol use					
Never	358 (49.4)	188 (25.8)	1.5	2	0.47
Monthly or less	91 (12.6)	37 (5.1)			
At least weekly	33 (4.6)	18 (2.5)			
Smoking					
Never smoke	453 (62.5)	231 (31.9)	0.3	1	0.55
Smoker	29 (4.0)	12 (1.7)			
Khat chewing					
Never	425 (58.6)	226 (31.2)	5.3	2	0.07
Monthly or less	24 (3.3)	10 (1.4)			
At least weekly	33 (4.6)	7 (1.0)			
Parent substance use					
Yes	113 (15.6)	47 (6.5)	1.6	1	0.21
No	369 (50.9)	196 (27.0)			
Substance user friend					
Yes	181 (25.0)	56 (7.7)	15.5	1	<0.001
No	301 (41.5)	187 (25.8)			
CAGE-AID score					
<2	408 (56.3)	219 (30.2)	4.1	1	0.04
>2	74 (10.2)	24 (3.3)			
Current substance use					
Yes	145 (20.0)	62 (8.6)	1.7	1	0.19
No	337 (46.5)	181 (25.0)			
Lifetime substance use					
No	311 (42.9)	174 (24.0)	3.7	1	0.06
Yes	171 (23.6)	69 (9.5)			
Age					
≤21 years	264 (36.4)	190 (26.4)	37.8	1	<0.001
>21 years	218 (30.1)	53 (7.3)			
Residence					
Rural	289 (39.8)	123 (17.0)	5.7	1	0.02
Urban	193 (26.6)	120 (16.6)			
Pocket money					
≤200 ETB	138 (19)	69 (9.5)	0.01	1	0.9
>200 ETB	344 (47.4)	174 (20.0)			

**Table 4:** Correlation of CGPA and selected variables among Wolaita Sodo University students, 2015.

Variables	CGPA	
	Correlation coefficient (*r*)	*p* value
Drinking on daily basis	−0.22	<0.001
Chew khat at least weekly	−0.23	<0.001
Smoking	−0.30	<0.001
Problematic use of substance (based on CAGE-AID)	−0.22	<0.001
Male gender	0.30	<0.001

**Table 5:** Linear regression for substance use and academic performance among students, 2015.

Variables	Beta	B (95% CI)
(Constant)	…	2.24 (0.89, 3.59)
Age of the student		
…	0.03	0.01 (−0.04, 0.06)
Sex		
Female		
Male	0.29	**0.35 (0.19, 0.52)** ^**∗∗∗**^
Marital status		
Single		
Ever married	−0.11	−0.27 (−0.58, 0.04)
In open relationship	−0.05	−0.08 (−0.27, 0.11)
Origin of residence		
Rural		
Urban	−0.17	**−0.19 (−0.32, −0.06)** ^**∗∗**^
Year of study		
…	0.02	0.01 (−0.06, 0.07)
Current smoking		
Yes	−0.19	**−0.27 (−0.46, −0.09)**
No		
Current khat chewing		
Never		
Monthly or less	−0.004	−0.01 (-0.20, 0.19)
At least weekly	−0.17	**−0.24 (−0.44, −0.04)** ^**∗**^
Having intimate friend who uses substance		
No		
Yes	−0.15	**−0.17 (−0.31, −0.03)** ^**∗**^
Parent substance use		
Yes	0.02	0.03 (−0.10, 0.16)
No		
Hostel service user		
Yes	0.08	0.09 (−0.06, 0.24)
No		
Happy for using substance		
Yes	−0.04	−0.04 (−0.18, 0.09)
No		
Any illness during the stay in the university		
Yes	0.09	0.18 (−0.04, 0.40)
No		
Interest in department (field of study)		
No	−0.01	−0.02 (−0.19, 0.15)
Yes		
Problematic substance use (CAGE-AID score ≥ 2)		
Yes	−0.09	−0.10 (−0.24, 0.04)
No		
Current substance use		
Yes	0.06	0.47 (−0.48, 1.43)
No		
Religion		
Orthodox		
Muslim	−0.03	−0.06 (−0.31, 0.18)
Protestant	−0.07	−0.09 (−0.24, 0.06)
Substance starting time		
Never		
Before joining WSU^§^		
At 1st year	−0.07	−0.09 (−0.25, 0.07)
Second year & above	−0.01	−0.02 (−0.19, 0.16)
Drinking alcohol		
Never		
Monthly	−0.08	−0.11 (−0.27, 0.05)
Weekly	−0.01	−0.02 (−0.21, 0.17)
Daily	−0.22	**−0.51 (−0.79, −0.23)** ^**∗∗∗**^

^§^Removed from the model. Dependent variable: CGPA, *R*^2^ = 0.45.

^*∗*^ *p* value < 0.05, ^*∗∗*^*p* value < 0.01, ^*∗∗∗*^*p* value < 0.001.

Beta: standardized coefficient; B: unstandardized coefficient.

## Abbreviations

ASSIST: The Alcohol, Smoking and Substance Involvement Screening Test
CAGE-AID: Cut down, Annoyed, Guilty, Eye opener-Adapted to Include Drugs
CGPA: Cumulative Grade Point Average
DSM-5: Diagnostic and Statistical manual of Mental disorders 5th edition
WHO: World Health Organization.

## Additional Points

*Limitations of the Study*. This study did not include substances other than tobacco products, alcohol, and khat. Interaction effect of these substances with academic achievement was also not assessed.
